# Supplementary material for: Medicine authentication technology as a counterfeit medicine-detection tool: a Delphi method study to establish expert opinion on manual medicine authentication technology in secondary care
Source: BMJ Open. 2017 May 6;7(5):e013838. doi: 10.1136/bmjopen-2016-013838 (PMC5777456; doi:10.1136/bmjopen-2016-013838)
Supplement: Author's manuscript [file bmjopen-2016-013838.draft_revisions.pdf]

BMJ Open

Medicine Authentication Technology as a Counterfeit Medicine Detection Tool : A Delphi Method Study to Establish Expert Opinion on Manual Medicine Authentication Technology in Secondary Care

Journal:	*BMJ Open*
Manuscript ID	bmjopen-2016-013838
Article Type:	Research
Date Submitted by the Author:	11-Aug-2016

Complete List of Authors:	Naughton, Bernard; Keele University Faculty of Health, Institute of Science and Technology in Medicine; University of Oxford Said Business School<br>Roberts, Lindsey; Academic Health Science Network, Medicines Optimisation<br>Dopson, Sue; University of Oxford, Said Business School<br>Brindley, David; University of Oxford, Paediatrics; University of Oxford, Said Buisness School<br>Chapman, Stephen; Keele University; University of Oxford, Department of Paediatrics

**Primary Subject Heading**:	Health policy
Secondary Subject Heading:	Global health, Health informatics, Public health, Health services research
Keywords:	Health informatics < BIOTECHNOLOGY & BIOINFORMATICS, Health policy < HEALTH SERVICES ADMINISTRATION & MANAGEMENT, PUBLIC HEALTH, Counterfeit drugs, Delphi technique

SCHOLARONE™ Manuscripts

For peer review only

Medicine Authentication Technology as a Counterfeit Medicine Detection Tool: A Delphi Method Study to Establish Expert Opinion on Manual Medicine Authentication Technology in Secondary Care

Naughton, Bernard (Doctoral Researcher) 1, 2, 3. Roberts Lindsey (Medicines Optimisation Network Manager) 4. Sue Dopson (Rhodes Trust Professor of Organisational Behaviour) 2. Brindley, David (Senior Research Fellow in Healthcare Translation) 2, 5-9. Chapman Stephen. (Professor of Prescribing Studies) 1,5.

1. Institute of Science and Technology in Medicine, Keele University, Stoke-on-Trent, UK.
2. Said Business School, University of Oxford, Oxford, UK.
3. Pharmacy Department, Oxford University Hospitals NHS Trust, Oxford, UK
4. Medicines Optimisation Clinical Network, Oxford Academic Health Science Network (AHSN), Oxford, UK.
5. Department of Pediatrics, University of Oxford, Oxford, UK.
6. The Oxford – UCL Centre for the Advancement of Sustainable Medical Innovation (CASMI), University of Oxford, Oxford, UK.
7. Centre for Behavioral Medicine, UCL School of Pharmacy, University College London, London, UK.
8. Harvard Stem Cell Institute, Cambridge, MA, USA.
9. USCF-Stanford Centre of Excellence in Regulatory Science and Innovation (CERSI), USA.

Author for correspondence: Bernard David Naughton, Said Business School, Oxford University, Park End Street, Oxford OX1 1 HP, bernard.naughton@sbs.ox.ac.uk, 0044(0)1865 614995.

DRAFT
For peer review only

For peer review only - http://bmjopen.bmj.com/site/about/guidelines.xhtml

Keywords: Delphi technique, Counterfeit drugs, Public Health, Health Policy, Medical Informatics.

Word count = 3,249

ABSTRACT

There has been a 34% increase in international drug counterfeiting in the last year, and a 50% increase since 2011 which demonstrates a medicines quality and safety concern internationally. The EU Falsified Medicines Directive (FMD) and US Drug Quality and Security Act (DQSA) both aim to tackle this problem through the serialization and verification of prescription only medicines using medicines authentication and verification technology. This study aims to establish expert opinion and potential improvements on medicines authentication technology. Consensus expert opinion has described the technology as quick and user friendly and suggested the inclusion of an audio signal amongst other recommendations to further support the detection of counterfeit medicines in secondary care (70% consensus, 0.9 SD).This paper aims to inform key opinion leaders and decision makers as to the positives and negatives of medicines authentication technology from an operators perspective and suggests the adjustments which may be required to improve the detection of counterfeit medicines and patient safety in secondary care across Europe and the US.

Strengths and limitations of this study

- This study is the first of its kind to obtain consensus regarding improvements necessary for medicines authentication technology, which is a pan-European approach to counterfeit medicine detection; driven by a European directive.
- The key consensus improvements for consideration have standard deviations ≤1.0

For peer review only - http://bmjopen.bmj.com/site/about/guidelines.xhtml

- This study focused on an emerging technology available in only one UK hospital, as such the available participants were limited
- This study would require implementation on a larger sample once suitable participants became available
- Although not uncommon, this Delphi method study contains two rounds

Introduction

Medicines counterfeiting is a global problem which requires international action.(1) There has been a 34% increase in international drug counterfeiting in the last year, and a 50% increase since 2011 (2). Countries around the world have developed a number of methods to detect counterfeit drugs which range from both lab based and portable Raman and Quadrupole spectroscopy in developed countries(3-6) to SMS messaging in low income and developing countries (7). A rapidly emerging method for the detection of counterfeit medicines internationally is the use of serialization and verification technology which has been adopted in the EU under the Falsified Medicines Directive (FMD)(8-10) and in the US under the Drug Quality and Security Act (DQSA) (11). The EU FMD prescribes the systematic serialisation at manufacture and authentication (the verification of a medicines authenticity and subsequent decommissioning) at the point of supply to the patient of prescription only medicines. Despite the 2019 legislative technology compliance deadline for pharmacies, dispensing GP practices and hospitals across Europe there is little qualitative evidence to support this international technological approach to counterfeit drug detection in practice. If implemented incorrectly this international change has the potential to cause considerable upset for healthcare providers (12,13).

This study aims to qualitatively evaluate and inform the optimisation of medicine authentication technology in secondary care. A group of ten secondary healthcare professionals with experience in medicines authentication were surveyed following a service evaluation study conducted

For peer review only - http://bmjopen.bmj.com/site/about/guidelines.xhtml

in 2015, using the Delphi method approach. The Delphi method approach, originally used as a systematic forecasting tool (14), has been increasingly used to gain consensus expert opinion and aid decision making in a variety of research areas . Delphi methodology provides clearer outcomes and recommendations than traditional surveys often produce, achieved by collecting responses and summarising responses until a consensus is achieved.

As part of the FMD, policy makers and key opinion leaders across Europe must come together to form National Medicines Verification Organisations (NMVO) and make financial and policy based decisions in this area. The outcomes of this study aims to educate decision makers as to the potentially useful technological advances which could be adopted or mandated as part of a national medicines verification system.

Methods

Twelve participants from a UK hospital pharmacy department with experience of using medicines authentication technology as part of a service evaluation project and satisfied the study inclusion and exclusion criteria were invited to take part in a Delphi method study. An implied consent model was used, with information regarding the nature of the study contained in the invitation email (14-18)(appendix 1:0) and repeated on the first page of the questionnaire. This information described the study and its voluntary nature. From a total population size of 12, 11 invitations were accepted. 1 staff member was unavailable due to sickness and one staff member was initially invited inappropriately, as they did not meet the inclusion criteria. 100% (n=10) of available eligible participants people responded (95% CI) (2% margin of error).

For peer review only - http://bmjopen.bmj.com/site/about/guidelines.xhtml

Table 1.0: Inclusion and Exclusion Criteria for Study Participants

Inclusion Criteria
Staff accredited in the professional checking process at the test site.<br>Staff with multiple experiences of using the authentication technology.<br>Staff willing to complete multiple surveys (12, 13.<br>Staff that have used the technology more than once.<br>Staff that have attended basic training as part of the service evaluation project on medicines authentication.
Exclusion criteria:
Staff that have used the system once or not at all.<br>Staffs that have not passed the trust checking accreditation test.

Prospective participants were asked to complete an electronic questionnaire (appendix 2) (14,19) with an estimated completion time of 15 minutes or less. Participants received a total of two questionnaires (17) (i.e. one for each round of iteration of the questionnaire). The initial invite was followed by a number of reminders at approximately 8 day intervals until completion. The question and response format included three different categories of questions (Table 2.0), a 7 point rating scale, a Likert scale and a descriptive or open ended response format. Likert scales were used to prioritise suggestions. In some cases the staff identified four suggestions and in some cases they identified five suggestions, therefore two Likert scales were employed.

Table 2.0: Question and response types

7-Point Rating Scale (r): This question format rates performance from 1 to 7, with '1' indicating a negative response and '7' indicating the most positive .The median response is also gathered for further consensus evaluation.
Likert Scale Response Format[9] (l) : This question style requires respondents to prioritise suggested improvements in terms of importance

For peer review only - http://bmjopen.bmj.com/site/about/guidelines.xhtml

from '1' to 4' or '1' to '5' with '1' indicating most important and '5' indicating least importance (median).
Descriptive/ Open-Ended Response Format (d): This response format does not require prioritization or rating, rather the analysis of responses generates a consensus descriptive answer for the group (median).

Consensus

The 7-point rating scale consensus was achieved when 70% of respondents selected either of two adjacent answers on a seven point rating scale (20) and the median score fell within either of the two consensus answers (21) (Table 3.0). In terms of the 5 point Likert scale response format (20) consensus will be achieved when 80% of the respondents select one of three adjacent answers in either direction i.e. whether participants classed the suggestion as "important" (1-3) or "not important" (3-5). On the 4-point Likert scale the same rules were applied, however, 'important' was classified as 1-2 and 'not important' was classified as 2-4 with consensus being achieved when 70% of respondents selected one of two answers in either direction (Table 3.0). In the case of a 5-point scale, the median must also be below 2.5, and in consideration of a 4-point scale the median must be below 2 to be considered 'important' (15) (Table 3.0).

In terms of the 5-point descriptive/ open-ended response format scale, consensus will be assessed dependent on the theme of the response e.g. if 80% of respondents selected one of three descriptive adjacent answers of the same sentiment (positive or negative) on a 5 point scale and the median score fell within the consensus category.

For peer review only - http://bmjopen.bmj.com/site/about/guidelines.xhtml

Table 3.0: Summary of Consensus

Question Type	Consensus (%)	Median response	Consensus Description
7-Point Rating Scale	70% agreement	Must fall within the consensus category	One of two adjacent answers
Likert Scale 5 point	80% agreement	<2.5 (Important)	One of three adjacent answers in either direction
Likert Scale 4 point	70% agreement	<2 (Important)	One of three adjacent answers in either direction
Descriptive/Open	80% agreement	Must fall within the consensus category	One of three adjacent answers of the same sentiment (positive or negative)

Summary of Survey Rounds

Round one involved three demographic questions, followed by a selection of closed questions relating to rating scales and performance and open questions requiring suggestions for a number of improvements, followed by descriptive questions to evaluate the quality of the survey. A selection of questions were built upon feedback from users, during a study which involved the use of authentication technology for an eight week period (reference submitted for publication in BMJ Open)

The administration of the second questionnaire was similar to round one; closed questions that achieved consensus were removed. Non-consensus questions were re-submitted to the audience in a rating scale style with further explanation.

For peer review only - http://bmjopen.bmj.com/site/about/guidelines.xhtml

Answers to the open ended questions in round one were thematically categorised and summarised to remove duplicate suggestions. In round two the experts were asked to answer further questions based on the most frequently occurring themes, which included likert scales or descriptive style questions formed as a direct result of participant suggestions during round one of the study or during the service evaluation study (reference submitted for publication in BMJ Open).The total number of suggestions per question varied between 4 and 5 suggestions which directly affected the number of options available in round two.

A valid consensus result was considered as 'achieved' when consensus had been met and the median scores also fell within the consensus group (Table 3.0). Suggestions that fall within these parameters and have a standard deviation of less than 1.0 were considered as the most relevant improvements for the authentication technology. There was no follow up with participants after study completion.

Results

A selection of questions which reached immediate consensus is described in Table 4.0 and 5.0. Answers in green boxes identify the results which reached consensus with a narrow standard deviation (less than 1.0). Answers in amber identify a consensus response with a larger standard deviation (greater than 1.0).

For peer review only - http://bmjopen.bmj.com/site/about/guidelines.xhtml

Table 4.0: A Summary of 7-Point Rating Scale Results

No	Question	Result	Standard Deviation (δ)
4	Based on your experience of the Medicines Authentication System (MAS), how would you rate its general speed on a scale of 1 to 7? (1 represents very slow and 7 represents very fast)	6/7	δ 0.75
5	Based on your experience of the MAS, how would you describe its usability on a scale of 1 to 7? (1 represents very difficult and 7 represents very easy)	6.5/7	δ 0.87
6	There were some system errors reported by the MAS users throughout the pilot. On a scale of 1 to 7 how often did you experience these types of errors? (1 represents never and 7 represents very often)(These errors may have included issues with reading the 2D barcode, duplication of the scan on screen or warnings such as "The system has no resources",” item can is invalid please scan product again" or "test product not found")	1/7	δ 0.67
Round 2			
7.	Question 4: How would you rate the impact of the MAS on the service you provide on a scale of 1-7 (where 1 represents very disruptive and 7 represents very helpful)?	4/7 (Not disruptive)	δ 0.94

For peer review only - http://bmjopen.bmj.com/site/about/guidelines.xhtml

Table 5.0: A summary of 4 and 5-Point Likert like scale results

No	Question	Result	Standard Deviation
**Round 1**			
7	The following are a list of reported, proposed improvements. Please rank them in order of importance (1-5)		
7(ii)	Change the medicine scanning list on screen to ensure the last scanned item appears on the top of the list	1 (Important)	δ 1.25
7(iv)	Review the pop up screens as the Red "Warning" screens could be mistaken for the common "Already dispensed here" screen.	2 (Important	δ 0.87
7 (v)	Incorporate "important information" pop-ups into the authentication system	2.5 (Important)	δ 1.25
**Round 2**			
5	During round one of this survey, further suggestions were made to improve the Medicines Authentication System (MAS) or the pilot. Please rank the suggested changes below in order of importance (1 being most important and 5 being least important).		
(ii)	Sounds could also be enabled to ensure	2.5	δ 1.2

	warnings/information are noticed (MAS)	(Important)	
6	During round one of this survey there were a variety of suggestions made to increase the Rate of Authentication (scanning). Valid suggestions were subdivided into three categories 1. Process Change 2.Technology change and 3.Education. In terms of Process Change please rank these suggestions in order of importance (with 1 being the most important and 5 being the least important)		
(I)	Make the symbol indicating an item that needs to be scanned larger/more visible (Process)	2.5 (Important)	δ 0.92
7	During round one of this survey there were a variety of suggestions made to increase the Rate of Authentication (scanning). Valid suggestions were subdivided into three categories 1. Process Change 2.Education and 3.Technology change In terms of Education and Technology change please rank these suggestions in order of importance (with 1 being the most important and 5 being the least important)		
(iv)	A system change that knows how many items	2.5	δ 1.2

DRAFT For peer review

For peer review only - http://bmjopen.bmj.com/site/about/guidelines.xhtml

	have been booked in and prescription is not able to be tracked out as verified until all medications have been authenticated (Technology Change)	(Important)	
8	During round one we explained that there have been occasions where products have been handed out despite showing a Pop-up Warning Box. We asked you to list three suggestions of how this occurrence might be reduced. Valid suggestions were subdivided into two categories 1.Education and 2.Technology change. In terms of Education please rank these suggestions in order of importance (with 1 being the most important and 4 being the least important)		
(I)	Encourage the dispenser/checker to take action on the warnings (Education)	2.0 (Important)	δ 1.0
9	During round one we explained that there have been occasions where products have been handed out despite showing a Pop-up Warning Box. We asked you to list three suggestions of how this occurrence might be reduced. Valid suggestions were subdivided into two categories 1.Education and 2.Technology change. In terms of Technology Change please rank these suggestions in order of importance		

DRAFT

For peer review

	(with 1 being the most important and 4 being the least important)		
(ii)	An audible alert to accompany the pop-up warning box (Technology)	1.00 (Important)	δ 0.9
(iii)	Making it mandatory to complete an "Action Taken" documentation process so that staff scanning are prompted to think about what the red warning means and be accountable for it (Technology)	2.00 (Important)	δ 0.94

The most important suggestions in this study are those which established consensus amongst the group (Table 3.0) with a narrow standard deviation. Improvements included reviewing the colour and information in warning pop up screens to ensure they were not mistaken for the "already dispensed here" pop up (2.0)(δ 0.87), encouraging the dispenser/checker to act on the warnings (2.0) (δ 1.0) (Education), including an audible alert to accompany the pop-up warning box (1.0) (δ 0.9) (Technology), and making it mandatory to complete an "Action Taken" documentation process to improve the quarantine process for potentially counterfeit, expired or recalled medicines. (Technology) (2.0)(δ 0.94).

Discussions and Recommendations

The serialization and authentication of medicines has been proposed in response to international regulation. Considering this technology will affect the entire EU and US it is important to gauge its current appropriateness and identify improvements in this valuable patient safety technology.

For peer review only - http://bmjopen.bmj.com/site/about/guidelines.xhtml

There were no concerns regarding the speed and usability of authentication technology raised during this study. There was limited impact on the daily activity of the staff and was classed as 'not disruptive'. Further qualitative research would be useful in understanding why the participants considered this technology as not disruptive.

The Delphi study identified a number of improvements including the importance of making clear differentiation between the various warning messaging to avoid misinterpretation. Participants identified concern with the similarity of warning messaging which may have had an impact on the decision to quarantine and may have contributed to the poor detection rates seen in previous publications relating to this study (reference submitted for publication to BMJ Open). This study recommends that authentication technology providers test their technology in a closed loop, real life environment prior to national implementation to ensure that operators respond appropriately to the messaging being displayed.

As with all changes to practice, adequate education and training is required. There was a basic presentation and protocol approach used in the service evaluation study (manuscript submitted for publication to BMJ Open) which may have contributed to inadequate authentication and detection rates due to lack of adequate support or perhaps due to the sheer volume of procedures and protocols that staff are required to adhere to(24). A practice change of this size may benefit from a structured training and revalidation package. If we refer to previous large information technology projects such as the implementation of the electronic patient record (EPR), we see a much more complicated electronic system with a structured training packages which largely includes, presentations, demonstrations, workshops, drop in sessions, protocols and guidance. A varied, informed and interactive approach, as used in the EPR project, is required to build operator background knowledge and to instill the clinical and legal importance of authenticating medicines as well as the impact of authentication on medicine detection rates.

For peer review only - http://bmjopen.bmj.com/site/about/guidelines.xhtml

A recent quantitative service evaluation study identified a disparity between medicines identified as requiring quarantine and those actually quarantined (manuscript submitted for publication to BMJ Open).This qualitative study demonstrated a consensus that a pop up warning message alone is not an adequate prompt in the heterogeneous hospital environment (10). The operators in this study are in agreement that a noise to indicate a medicine as requiring quarantine is required. Not only would this remind the operator to act but it would also bring attention to the entire team that a medicine requiring quarantine had been identified, generating peer pressure to act on the warning.

Participants emphasised the importance of an 'Action taken' documentation process. Operators relied on recording the medicine for quarantine on a proforma located beside the terminal; their view was that this could be improved with the inclusion of an 'Action Taken' function incorporated into the authentication technology software. This may facilitate a reporting system which would benefit managerial monitoring of falsified, counterfeit, recalled or expired medicines. This would also allow quarantine staff to tally medicines physically quarantined with medicines identified by the technology as requiring quarantine.

Each hospital in the NHS varies slightly depending on the services it provides, which makes context an important factor in technology implementation. Awareness of internal and external context, in the planning stages of implementation will play an increasingly important role in the introduction of new technologies into the healthcare setting (22,23). Further qualitative research is required to understand how context management and change management can facilitate successful technological projects in the NHS. One such contextual factor may include incentives to authenticate medicines, the use of reimbursement codes within the 2D data matrix codes is hypothesized to help the authentication rate of medicines, a practice seen in the Belgian community pharmacy setting. There will be a legal mandate to authenticate medicines, however incentives such as

For peer review only - http://bmjopen.bmj.com/site/about/guidelines.xhtml

reimbursement upon authentication may prove to augment technological and legal compliance.

These results should inform key opinion leaders, policy makers and technology manufacturers as to potential authentication technology improvements. Considering the limited evidence to support medicines authentication the outcomes of this study should service decision makers in their discussions surrounding the selection of medicines authentication technology providers.

CONTRIBUTION STATEMENT

BN and DB were responsible for study conception, BN, DB, LR, SD and SC were responsible for planning, BN was responsible for data collection and scripting, BN and LR were responsible for data analysis. BN, DB, LR, SD and SC were responsible for the reviewing of this manuscript.

COMPETING INTERISTS

The content outlined herein represents the individual opinions of the authors and may not necessarily represent the viewpoints of their employers. D.A.B is an employee and/or stockholders in Aegate Ltd (Melbourn, UK) that is a provider of medicines authentication services. D.A.B. is also a stockholder in Translation Ventures Ltd. (Charlbury, Oxfordshire, UK) and IP asset ventures. D.A.B. is subject to the CFA Institute’s Codes, Standards, and Guidelines, and as such, this author must stress that this piece is provided for academic interest only and must not be construed in any way as an investment recommendation. B.N is currently not, but has previously been a consultant of Aegate limited. Additionally, at time of publication, D.A.B. and the organizations with which he is affiliated may or may not have agreed and/or pending funding commitments from the organizations named herein.

For peer review only - http://bmjopen.bmj.com/site/about/guidelines.xhtml

FUNDING

This study was funded by Keele University, Oxford University and Aegate limited via an unrestricted educational grant.

DATA SHARING STATEMENT

Raw research data is available via Dryad

REFERENCES

1. Mackey TK, Liang BA. Improving global health governance to combat counterfeit medicines: a proposal for a UNODC-WHO-Interpol trilateral mechanism. BMC Med. 2013;11(1):233.

2. PSI-Inc.org [Internet]. [cited 2016 Jun 9]. Available from: http://www.psi-inc.org/incidentTrends.cfm

3. Hajjou M, Qin Y, Bradby S, Bempong D, Lukulay P. Assessment of the performance of a handheld Raman device for potential use as a screening tool in evaluating medicines quality. J Pharm Biomed Anal. 2013 Feb 23;74:47–55.

4. Mbinze JK, Sacré P-Y, Yemoa A, Mavar Tayey Mbay J, Habyalimana V, Kalenda N, et al. Development, validation and comparison of NIR and Raman methods for the identification and assay of poor-quality oral quinine drops. J Pharm Biomed Anal. 2015;111:21–7.

5. Sacré P-Y, Deconinck E, Saerens L, De Beer T, Courselle P, Vancauwenberghe R, et al. Detection of counterfeit Viagra® by Raman microspectroscopy imaging and multivariate analysis. J Pharm Biomed Anal. 2011 Sep 10;56(2):454–61.

6. Chen C, Zhang F, Barras J, Althoefer K, Bhunia S, Mandal S. Authentication of medicines using nuclear quadrupole resonance spectroscopy. IEEE/ACM Trans Comput Biol Bioinform. 2015;1–1.

7. Yeebo Y. The African Startup Using Phones to Spot Counterfeit Drugs [Internet]. Bloomberg.com. [cited 2016 Feb 17]. Available from: http://www.bloomberg.com/news/features/2015-07-31/the-african-startup-using-phones-to-spot-counterfeit-drugs

8. Book Excerpt: Fundamentals of EU Regulatory Affairs, Seventh Edition, Chapter 8: European Union Falsified Medicines Directive: Requirements and Implications for Multi-Stakeholder Healthcare Delivery | RAPS [Internet]. [cited 2016 Jan 25]. Available from: http://www.raps.org/Regulatory-Focus/RAPS-Latest/2015/12/18/23808/Book-Excerpt-Fundamentals-of-EU-Regulatory-Affairs-Seventh-Edition-Chapter-8-European-Union-Falsified-Medicines-Directive-Requirements-and-Implications-for-Multi-Stakeholder-Healthcare-Delivery/

For peer review only - http://bmjopen.bmj.com/site/about/guidelines.xhtml

9. COMMISSION DELEGATED REGULATION (EU) 2016/ 161 - of 2 October 2015 - supplementing Directive 2001/ 83/ EC of the European Parliament and of the Council by laying down detailed rules for the safety features appearing on the packaging of medicinal products for human use - http://eur-lex.europa.eu/legal-content/EN/TXT/PDF/?uri=OJ:JOL_2016_032_R_0001&from=EN [Internet]. [cited 2016 May 3]. Available from: http://eur-lex.europa.eu/legal-content/EN/TXT/PDF/?uri=OJ:JOL_2016_032_R_0001&from=EN

10. Directive 2011/62/EU of the European Parliament and of the Council of 8 June 2011 amending Directive 2001/83/EC on the Community code relating to medicinal products for human use, as regards the prevention of the entry into the legal supply chain of falsified medicinal productsText with EEA relevance - dir_2011_62_en.pdf [Internet]. [cited 2015 Jul 9]. Available from: http://ec.europa.eu/health/files/eudralex/vol-1/dir_2011_62/dir_2011_62_en.pdf

11. Upton F. H.R.3204 - 113th Congress (2013-2014): Drug Quality and Security Act [Internet]. 2013 [cited 2016 Feb 8]. Available from: https://www.congress.gov/bill/113th-congress/house-bill/3204

12. Naughton B, Vadher B, Smith J, Smith G, Chapman S, Dopson S, et al. EU Falsified Medicines Directive mandatory requirements for secondary care: A concise review. J Generic Med Bus J Generic Med Sect [Internet]. 2016 Apr 21 [cited 2016 May 18]; Available from: http://jgm.sagepub.com/lookup/doi/10.1177/1741134316643358

13. Naughton BD, Smith JA, Brindley DA. Establishing good authentication practice (GAP) in secondary care to protect against falsified medicines and improve patient safety. Eur J Hosp Pharm Sci Pract. 2016 Mar;23(2):118–20.

14. Rowe G, Wright G. Expert Opinions in Forecasting: The Role of the Delphi Technique. In: Armstrong JS, editor. Principles of Forecasting [Internet]. Springer US; 2001 [cited 2015 Nov 9]. p. 125–44. (International Series in Operations Research & Management Science). Available from: http://link.springer.com/chapter/10.1007/978-0-306-47630-3_7

15. The Delphi Technique: Making Sense Of Consensus - v12n10.pdf [Internet]. [cited 2015 Nov 9]. Available from: http://pareonline.net/pdf/v12n10.pdf

16. Planning and Conducting Needs Assessments | SAGE Publications Ltd [Internet]. [cited 2016 May 25]. Available from: https://uk.sagepub.com/en-gb/eur/planning-and-conducting-needs-assessments/book5003

17. Adler M, Ziglio E. Gazing Into the Oracle: The Delphi Method and Its Application to Social Policy and Public Health. Jessica Kingsley Publishers; 1996. 270 p.

18. Predicting the Future: Have you considered using the Delphi Methodology? [Internet]. [cited 2015 Nov 9]. Available from: http://www.joe.org/joe/1997october/tt2.php/index.php

19. Group techniques for program planning□: a guide to nominal group and delphi processes | Clc [Internet]. [cited 2015 Nov 9]. Available from: http://library.wur.nl/WebQuery/clc/347478

For peer review only - http://bmjopen.bmj.com/site/about/guidelines.xhtml

20. Ulschak FL. Human resource development: the theory and practice of need assessment. Reston Pub. Co.; 1983. 328 p.

21. Green P. The Content of a College-Level Outdoor Leadership Course. 1982 Mar [cited 2015 Nov 9]; Available from: http://eric.ed.gov/?id=ED276546

22. Dopson S, Fitzgerald L, Ferlie E. Understanding Change and Innovation in Healthcare Settings: Reconceptualizing the Active Role of Context. J Change Manag. 2008 Dec;8(3-4):213-31.

23. Robert G, Fulop N. The role of context in successful improvement. Perspect Context Sel Essays Considering Role Context Success Qual Improv Lond Health Found [Internet]. 2014 [cited 2016 Jul 4];31. Available from: http://www.health.org.uk/sites/default/files/PerspectivesOnContextRobertFulopTheRoleOfContextInSuccessfulImprovement.pdf

24. Carthey J, Walker S, Deelchand V, Vincent C, Griffiths WH. Breaking the rules: understanding non-compliance with policies and guidelines. BMJ. 2011 Sep 13;343(sep13 3):d5283-d5283.

DRAFT
For peer review only

For peer review only - http://bmjopen.bmj.com/site/about/guidelines.xhtml

BMJ Open

Medicine Authentication Technology as a Counterfeit Medicine Detection Tool : A Delphi Method Study to Establish Expert Opinion on Manual Medicine Authentication Technology in Secondary Care

Journal:	*BMJ Open*
Manuscript ID	bmjopen-2016-013838.R1
Article Type:	Research
Date Submitted by the Author:	03-Nov-2016
Complete List of Authors:	Naughton, Bernard; Keele University Faculty of Health, Institute of Science and Technology in Medicine; University of Oxford Said Business School<br>Roberts, Lindsey; Academic Health Science Network, Medicines Optimisation<br>Dopson, Sue; University of Oxford, Said Business School<br>Brindley, David; University of Oxford, Paediatrics; University of Oxford, Said Buisness School<br>Chapman, Stephen; Keele University; University of Oxford, Department of Paediatrics
**Primary Subject Heading**:	Health policy
Secondary Subject Heading:	Global health, Health informatics, Public health, Health services research
Keywords:	Health informatics < BIOTECHNOLOGY & BIOINFORMATICS, Health policy < HEALTH SERVICES ADMINISTRATION & MANAGEMENT, PUBLIC HEALTH, Counterfeit drugs, Delphi technique

SCHOLARONE™
Manuscripts

For peer review only

Medicine Authentication Technology as a Counterfeit Medicine Detection Tool: A Delphi Method Study to Establish Expert Opinion on Manual Medicine Authentication Technology in Secondary Care

Naughton, Bernard (Doctoral Researcher) 1, 2, 3. Roberts Lindsey (Medicines Optimisation Network Manager) 4. Sue Dopson (Rhodes Trust Professor of Organisational Behaviour) 2. Brindley, David (Senior Research Fellow in Healthcare Translation) 2, 5-9. Chapman Stephen. (Professor of Prescribing Studies) 1,5.

1. Institute of Science and Technology in Medicine, Keele University, Stoke-on-Trent, UK.
2. Said Business School, University of Oxford, Oxford, UK.
3. Pharmacy Department, Oxford University Hospitals NHS Trust, Oxford, UK
4. Medicines Optimisation Clinical Network, Oxford Academic Health Science Network (AHSN), Oxford, UK.
5. Department of Pediatrics, University of Oxford, Oxford, UK.
6. The Oxford – UCL Centre for the Advancement of Sustainable Medical Innovation (CASMI), University of Oxford, Oxford, UK.
7. Centre for Behavioral Medicine, UCL School of Pharmacy, University College London, London, UK.
8. Harvard Stem Cell Institute, Cambridge, MA, USA.
9. USCF-Stanford Centre of Excellence in Regulatory Science and Innovation (CERSI), USA.

Author for correspondence: Bernard David Naughton, Said Business School, Oxford University, Park End Street, Oxford OX1 1 HP, bernard.naughton@sbs.ox.ac.uk, 0044(0)1865 614995.

For peer review only - http://bmjopen.bmj.com/site/about/guidelines.xhtml

Keywords: Delphi technique, Counterfeit drugs, Public Health, Health Policy, Medical Informatics.

Word count = 5,259

ABSTRACT

Objectives: This study aims to establish expert opinion and potential improvements for the Falsified Medicines Directive mandated medicines authentication technology.

Design and Intervention: A two round Delphi method study using an online questionnaire.

Setting: Large NHS foundation trust teaching hospital

Participants: Secondary care pharmacists and accredited checking technicians.

Primary outcome measures: Seven-point rating scale answers which reached a consensus of 70-80% with a standard deviation of less than 1.0. Likert scale questions which reached a consensus of 70-80%, a standard deviation of less than 1.0 and classified as important according to study criteria.

Results: Consensus expert opinion has described database cross-checking technology as quick and user friendly and suggested the inclusion of an audio signal to further support the detection of counterfeit medicines in secondary care (70% consensus, 0.9 SD) other important consensus with a standard deviation of less than 1.0 included reviewing the colour and information in warning pop up screens to ensure they were not mistaken for the “already dispensed here” pop up, encouraging the dispenser/checker to act on the warnings and making it mandatory to complete an "Action Taken" documentation process to improve the quarantine of potentially counterfeit, expired or recalled medicines.

For peer review only - http://bmjopen.bmj.com/site/about/guidelines.xhtml

Conclusions:

This paper informs key opinion leaders and decision makers as to the positives and negatives of medicines authentication technology from an operator's perspective and suggests the adjustments which may be required to improve operator compliance and the detection of counterfeit medicines in the secondary care sector.

Strengths and limitations of this study

- This study is the first of its kind to obtain consensus regarding improvements necessary for medicines authentication technology, which is a Pan-European approach to counterfeit medicine detection; driven by a European directive.
- The key consensus improvements for consideration have narrow standard deviations of ≤1.0
- This study focused on a serialization and authentication technology provided by one of the three EMVO 'Blueprint providers'.
- At the time of research, authentication technology was available in only one UK hospital, as such the available participants were limited
- This study would require implementation on a larger sample once suitable participants became available
- Although not uncommon, this Delphi method study contains two rounds

For peer review only - http://bmjopen.bmj.com/site/about/guidelines.xhtml

Introduction

According to the European Commission a falsified medicine is a fake medicine that passes itself off as a real authorized medicine (1), according to the FDA a counterfeit medicine is a 'fake medicine' that may be contaminated or contain the wrong or no active ingredient. It could have the right active ingredient but at the wrong dose (2). According to international intellectual property law a 'counterfeit' is one that infringes a trademark by bearing an identical or near identical mark.(3) In contrast the world health organization uses the term SSFFC which stands for substandard, spurious, falsely labelled, falsified and counterfeit medicinal products(4). The EMA term of 'falsified medicine' is used in relation to the falsified medicines directive (FMD) while the FDA term of counterfeit medicine is used in relation to the drug supply chain security act (DSCSA).

Medicines counterfeiting is a global problem which requires international action (5). According to the pharmaceutical security institute (PSI) there has been a 34% increase in international drug counterfeiting, illegal diversion and theft in the last year, and a 51% increase since 2011 (6). Countries around the world have developed a number of methods to detect counterfeit drugs which range from both lab based and portable Raman and Quadrupole spectroscopy in developed countries(7-10) to SMS messaging in low income and developing countries (11).

This study assesses serialization and verification technology, the most rapidly emerging method for detecting counterfeit medicines. This technology has been adopted in the EU under the Falsified Medicines Directive (FMD)(12-14) and in the US under the Drug Supply Chain Security Act (DSCSA) (15). Authentication relates to the verification of a medicines authenticity and subsequent decommissioning using database cross checking of the medicines unique 12-digit serial code, at the point of supply to the patient. While verification refers to the scanning of a

For peer review only - http://bmjopen.bmj.com/site/about/guidelines.xhtml

medicinal product to identify its status in terms of falsification, expiry or recalled status without decommissioning. The EU FMD prescribes the systematic serialisation at manufacture and authentication at the point of supply to the patient while the DSCSA requires the verification of medicinal product at each change of ownership without verification or decommissioning at the point of supply to the patient.

The European Medicines Verification Organisation (EMVO) have described the core principles of authentication technology and have appointed three blueprint authentication technology providers for Europe. These providers are Aegate Holdings limited, Arvato Systems GmbH and Solidsoft Reply. Each of these providers are expected to meet the minimum requirements as set by the EMVO (16). As only one authentication technology, provided by Aegate holdings limited, was operational at the time of research it was chosen to be integrated into a live secondary care hospital environment as part of a service evaluation study. Upon the scanning and subsequent detection of a recalled, expired or potentially counterfeit medicine this technology presents the user with red or amber pop up messages which identifies a product as requiring quarantine. Medicines authenticated which do not require quarantine do not generate a pop up but instead generate a 'non action' symbol. Upon integration staff were trained via presentation and demonstration and also issued with an authentication protocol which detailed step by step instructions on how to authenticate medicines.

The key results from the service evaluation study related to authentication rates and detection rates of the technology. Although the technical detection rate of the technology was 100% and the response rate of the technology was less than 300 milliseconds (as mandated by the FMD)(14)(13) not all medicines requiring authentication were authenticated and of those authenticated not all of those that generated a warning pop-up were quarantined.(17) Following the service evaluations study this Delphi

For peer review only - http://bmjopen.bmj.com/site/about/guidelines.xhtml

method study was conducted to identify the reasons for poor authentication and detection rates and identify improvements.

Despite the 2019 legislative technology compliance deadline for pharmacies, dispensing general practitioner (GP) practices and hospitals across Europe there is little qualitative evidence to support this international technological approach to counterfeit drug detection in practice. If implemented incorrectly this international change has the potential to cause considerable upset for healthcare providers. This study aims to qualitatively evaluate and inform the optimisation of medicine authentication technology in secondary care.

A group of ten secondary healthcare professionals with experience in medicines authentication were surveyed following a service evaluation study conducted in 2015, using the Delphi method approach. The Delphi method approach, originally used as a systematic forecasting tool (18)(19), has been increasingly used to gain consensus expert opinion and aid decision making in a variety of research areas . Delphi methodology provides clearer outcomes and recommendations than traditional surveys often produce, achieved by collecting responses and summarising responses until a consensus is achieved.

As part of the FMD, policy makers and key opinion leaders across Europe must come together to form National Medicines Verification Organisations (NMVO). Each NMVO will make decisions regarding authentication technology providers which may relate to functionality, speed of data response, usability and technology limitations. The study aims to identify the positives of the incoming technology and educate decision makers as to the potentially useful technological changes that could be adopted or mandated as part of the incumbent medicines authentication technology.

For peer review only - http://bmjopen.bmj.com/site/about/guidelines.xhtml

Methods

Twelve participants from a UK hospital pharmacy department with experience of using medicines authentication technology as part of a service evaluation project and satisfied the study inclusion and exclusion criteria were invited to take part in a Delphi method study. An implied consent model was used, with information regarding the nature of the study contained in the invitation email (18-22) and repeated on the first page of the questionnaire. This information described the study and its voluntary nature. From a total population size of 12, 11 invitations were accepted. 1 staff member was unavailable due to sickness and one staff member was initially invited inappropriately, as they did not meet the inclusion criteria. 100% (n=10) of available eligible participants responded (95% CI) (2% margin of error).

Table 1.0: Inclusion and Exclusion Criteria for Study Participants

Inclusion Criteria
Staff accredited in the professional checking process at the test site*.<br>Staff with multiple experiences of using the authentication technology.<br>Staff willing to complete multiple surveys<br>Staff that have used the technology more than once.<br>Staff that have attended basic training as part of the service evaluation project on medicines authentication.
Exclusion criteria:
Staff that have used the system once or not at all.<br>Staff that have not passed the trust checking accreditation test.

*Staff were a mixture of GPHC registered pharmacists and qualified accuracy checking technicians (nationally recognized qualification)

Prospective participants were asked to complete an electronic questionnaire (18,23) with an estimated completion time of 15 minutes or less.

For peer review only - http://bmjopen.bmj.com/site/about/guidelines.xhtml

Participants received a total of two questionnaires (21) (i.e. one for each round of iteration of the questionnaire). The initial invite was followed by a number of reminders at approximately 8 day intervals until completion. The question and response format included three different categories of questions (Table 2.0), a 7-point rating scale, a Likert scale and a descriptive or open ended response format. Likert scales were used to prioritise suggestions. In some cases, the staff identified four suggestions and in some cases they identified five suggestions, therefore two Likert scales were employed.

Table 2.0: Question and response types

7-Point Rating Scale (r): This question format rates performance from 1 to 7, with '1' indicating a negative response and '7' indicating the most positive .The median response was also gathered for further consensus evaluation.
Likert Scale Response Format[9] (l) : This question style requires respondents to prioritise suggested improvements in terms of importance from '1' to 4' or '1' to '5' with '1' indicating most important and '5' indicating least importance (median).
Descriptive/ Open-Ended Response Format (d): This response format does not require prioritization or rating, rather the analysis of responses generates a consensus descriptive answer for the group (median).

Consensus

The 7-point rating scale consensus was achieved when 70% of respondents selected either of two adjacent answers on a seven point rating scale (24) and the median score fell within either of the two consensus answers (25) (Table 3.0). In terms of the 5 point Likert scale response format (24) consensus was achieved when 80% of the respondents selected one of three adjacent answers in either direction i.e. whether participants classed the suggestion as "important" (1-3) or "not important" (3-5). On the 4-point

For peer review only - http://bmjopen.bmj.com/site/about/guidelines.xhtml

Likert scale the same rules were applied, however, 'important' was classified as 1-2 and 'not important' was classified as 2-4 with consensus being achieved when 70% of respondents selected one of two answers in either direction (Table 3.0). In the case of a 5-point scale, the median must also have been below 2.5, and in consideration of a 4-point scale the median must also have been below 2 to be considered 'important' (19) (Table 3.0).

In terms of the 5-point descriptive/ open-ended response format scale, consensus was assessed dependent on the theme of the response e.g. if 80% of respondents selected one of three descriptive adjacent answers of the same sentiment (positive or negative) on a 5-point scale and the median score fell within the consensus category.

The planning of this study was based largely on the highly cited Hsu and Sandford paper from 2007 which describes evidence for successful Delphi studies such as number of survey rounds, consensus figures and thematic analysis (19). Delphi method consensus varied across studies and opinions differs on a suitable percentage measure. This study compliments those conducted by Green in 1982 (24) and Ulschak in 1983 (25) (in Hsu and Sandford in 2007) (19)which promote a consensus of 70% and 80% respectively. The default consensus in this study was 70% with 80% used where possible.

Standard deviation was chosen as a method to measure central tendencies with an arbitrary <1.0 SD used to supplement the consensus. This quantified the spread of responses across the entire group.

Table 3.0: Summary of Consensus

Question Type	Consensus (%)	Median response	Consensus Description
7-Point Rating Scale	70% agreement	Must fall within the consensus	One of two adjacent answers

For peer review only - http://bmjopen.bmj.com/site/about/guidelines.xhtml

		category	
Likert Scale 5 point	80% agreement	<2.5 (Important)	One of three adjacent answers in either direction
Likert Scale 4 point	70% agreement	<2 (Important)	One of three adjacent answers in either direction
Descriptive/Open	80% agreement	Must fall within the consensus category	One of three adjacent answers of the same sentiment (positive or negative)

Summary of Survey Rounds

Round one involved three demographic questions, followed by a selection of closed questions relating to rating scales and performance and open questions requiring suggestions for a number of improvements, followed by descriptive questions to evaluate the quality of the survey. A selection of questions were built upon feedback from users, during a study which involved the use of authentication technology for an eight-week period (17) The administration of the second questionnaire was similar to round one; closed questions that achieved consensus were removed. Non-consensus questions were re-submitted to the audience in a rating scale style with further explanation.

Answers to the open ended questions in round one were thematically categorised and summarised to remove duplicate suggestions. In round two the experts were asked to answer further questions based on the most frequently occurring themes, which included Likert scales or descriptive style questions formed as a direct result of participant suggestions during round one of the study or during the service evaluation study (17).The total number of suggestions per question varied between 4 and 5 suggestions which directly affected the number of options available in round two.

For peer review only - http://bmjopen.bmj.com/site/about/guidelines.xhtml

A valid consensus result was considered as 'achieved' when consensus had been met and the median scores also fell within the consensus group (Table 3.0). Suggestions that fell within these parameters and had a standard deviation of less than 1.0 were considered as the most relevant improvements for the authentication technology. There was no follow up with participants after study completion.

Results

A selection of questions which reached immediate consensus is described in Table 4.0 and 5.0. Answers in green boxes identify the results which reached consensus with a narrow standard deviation (less than 1.0). Answers in amber identify a consensus response with a larger standard deviation (greater than 1.0).

Table 4.0: A Summary of 7-Point Rating Scale Results

No	Question	Result	Standard Deviation (SD)
4	Based on your experience of the Medicines Authentication System (MAS), how would you rate its general speed on a scale of 1 to 7? (1 represents very slow and 7 represents very fast)	6/7	SD 0.75
5	Based on your experience of the MAS, how would you describe its usability on a scale of 1 to 7? (1 represents very difficult and 7 represents very easy)	6.5/7	SD 0.87
6	There were some system errors reported by the MAS users throughout the pilot. On a scale of 1 to 7 how often did you experience these	1/7	SD 0.67

For peer review only - http://bmjopen.bmj.com/site/about/guidelines.xhtml

	types of errors? (1 represents never and 7 represents very often) (These errors may have included issues with reading the 2D barcode, duplication of the scan on screen or warnings such as "The system has no resources",” item can is invalid please scan product again" or "test product not found")		
Round 2			
7.	Question 4: How would you rate the impact of the MAS on the service you provide on a scale of 1-7 (where 1 represents very disruptive and 7 represents very helpful)?	4/7 (Not disruptive)	SD 0.94

Table 5.0: A summary of 4 and 5-Point Likert like scale results

No	Question	Result	Standard Deviation (SD)
**Round 1**			
7	The following are a list of reported, proposed improvements. Please rank them in order of importance (1-5)		
7(ii)	Change the medicine scanning list on screen to ensure the last scanned item appears on the top of the list	1 (Important)	SD 1.25

For peer review only - http://bmjopen.bmj.com/site/about/guidelines.xhtml

7(iv)	Review the pop up screens as the Red "Warning" screens could be mistaken for the common "Already dispensed here" screen.	2 (Importa nt	SD 0.87
7 (v)	Incorporate "important information" pop-ups into the authentication system	2.5 (Importa nt)	SD 1.25
**Round 2**			
5	During round one of this survey, further suggestions were made to improve the Medicines Authentication System (MAS) or the pilot. Please rank the suggested changes below in order of importance (1 being most important and 5 being least important).		
(ii)	Sounds could also be enabled to ensure warnings/information are noticed (MAS)	2.5 (Importa nt)	SD 1.2
6	During round one of this survey there were a variety of suggestions made to increase the Rate of Authentication (scanning). Valid suggestions were subdivided into three categories 1. Process Change 2.Technology change and 3.Education. In terms of Process Change please rank these suggestions in order of importance (with 1 being the most important and 5 being the least important)		

For peer review only - http://bmjopen.bmj.com/site/about/guidelines.xhtml

(I)	Make the symbol indicating an item that needs to be scanned larger/more visible (Process)	2.5 (Important)	SD 0.92
7	During round one of this survey there were a variety of suggestions made to increase the Rate of Authentication (scanning). Valid suggestions were subdivided into three categories 1. Process Change 2. Education and 3. Technology change In terms of Education and Technology change please rank these suggestions in order of importance (with 1 being the most important and 5 being the least important)		
(iv)	A system change that knows how many items have been booked in and prescription is not able to be tracked out as verified until all medications have been authenticated (Technology Change)	2.5 (Important)	SD 1.2
8	During round one we explained that there have been occasions where products have been handed out despite showing a Pop-up Warning Box. We asked you to list three suggestions of how this occurrence might be reduced. Valid suggestions were subdivided into two categories 1. Education and 2. Technology change. In terms of Education please rank these		

For peer review only - http://bmjopen.bmj.com/site/about/guidelines.xhtml

	suggestions in order of importance (with 1 being the most important and 4 being the least important)		
(I)	Encourage the dispenser/checker to take action on the warnings (Education)	2.0 (Important)	SD 1.0
9	During round one we explained that there have been occasions where products have been handed out despite showing a Pop-up Warning Box. We asked you to list three suggestions of how this occurrence might be reduced. Valid suggestions were subdivided into two categories 1. Education and 2. Technology change. In terms of Technology Change please rank these suggestions in order of importance (with 1 being the most important and 4 being the least important)		
(ii)	An audible alert to accompany the pop-up warning box (Technology)	1.00 (Important)	SD 0.9
(iii)	Making it mandatory to complete an "Action Taken" documentation process so that staff scanning are prompted to think about what the red warning means and be accountable for it (Technology)	2.00 (Important)	SD 0.94

For peer review only - http://bmjopen.bmj.com/site/about/guidelines.xhtml

The most important suggestions in this study are those which established consensus amongst the group (Table 3.0) with a narrow standard deviation. Improvements included reviewing the colour and information in warning pop up screens to ensure they were not mistaken for the “already dispensed here” pop up (2.0)(SD 0.87), encouraging the dispenser/checker to act on the warnings (2.0) (SD 1.0) (Education), including an audible alert to accompany the pop-up warning box (1.0) (SD 0.9) (Technology), and making it mandatory to complete an "Action Taken" documentation process to improve the quarantine process for potentially counterfeit, expired or recalled medicines. (Technology) (2.0) (SD 0.94).

Discussions and Recommendations

The serialization and authentication of medicines has been proposed in response to international regulation. Considering that serialization and verification with or without authentication will affect EU hospital pharmacies and is likely to affect US hospitals wishing to wholesale supply, it is important to gauge its current appropriateness and identify improvements in this valuable patient safety technology.

There were no concerns regarding the speed and usability of authentication technology raised during this study. There was limited impact on the daily activity of the staff and was classed as ‘not disruptive’. Further qualitative research would be useful in understanding why the participants considered this technology as not disruptive.

The Delphi study identified a number of suggestions to improve authentication and detection rates including the importance of making clear differentiation between the various warning messaging to avoid misinterpretation. Participants identified concern with the similarity of warning messaging which may have had an impact on the decision to quarantine and may have contributed to the suboptimal detection rates seen in previous publications relating to the service evaluation study (17).

For peer review only - http://bmjopen.bmj.com/site/about/guidelines.xhtml

This study recommends that authentication technology providers test their technology in a closed loop, real life environment prior to national implementation to ensure that operators respond appropriately to the messaging being displayed.

As with all changes to practice, adequate education and training is required. There was a basic presentation and protocol approach used in the service evaluation study (17) which may have contributed to inadequate authentication and detection rates due to lack of adequate support or perhaps due to the sheer volume of procedures and protocols that staff are required to adhere to. A practice change of this size may benefit from a structured training and revalidation package. If we refer to previous large information technology projects such as the implementation of the electronic patient record (EPR), we often saw a much more complicated electronic system with a structured training packages which largely includes, presentations, demonstrations, workshops, drop in sessions, protocols and guidance. A varied, informed and interactive approach, as used in the EPR project, is required to build operator background knowledge and to instill the clinical and legal importance of authenticating medicines as well as the impact of authentication on medicine detection rates.

The recent quantitative service evaluation study identified a disparity between medicines identified as requiring quarantine and those actually quarantined (17).This qualitative study demonstrated a consensus that a pop up warning message alone is not an adequate prompt in the heterogeneous hospital environment (10). The operators in this study are in agreement that a noise to indicate a medicine as requiring quarantine is required. Not only would this remind the operator to act but it would also bring attention to the entire team that a medicine requiring quarantine had been identified, generating peer pressure to act on the warning.

Participants emphasised the importance of an 'Action taken' documentation process. Operators relied on recording the medicine for quarantine on a proforma located beside the terminal; their view was that this could be improved with the inclusion of an 'Action Taken' function incorporated into the authentication technology software. In a systematic

For peer review only - http://bmjopen.bmj.com/site/about/guidelines.xhtml

review study by Shojania et al the action taken alert has been identified as potentially more effective than a 'non action' alert ,(26) demonstrated in a small group. This 'action taken' approach may facilitate an improved dection rate and may also support a reporting system which would benefit managerial monitoring of falsified, counterfeit, recalled or expired medicines within a department. This would also allow staff responsible for product quarantine to tally medicines physically quarantined with medicines identified by the technology as requiring quarantine.

Information technology systems such as electronic prescribing or in this case medicines authentication are relatively new approaches to optimise healthcare information. Shojania et al (26) demonstrates that evidence to support computer alerts is currently limited. In relation to electronic prescribing, Shojania concludes by stating 'Further research must identify design features and contextual factors consistently associated with larger improvements in provider behaviour if computer reminders are to succeed on more than a trial and error basis'. Further research is also required in relation to medicines authentication technology to identify the approaches which facilitate operational compliance. Hospitals in the NHS varies slightly depending on the services they provide, which makes context an important factor in technology success. (27). Following on from the remarks made by Shojania et al further research is required to understand how contextual factors can facilitate successful technological projects in the NHS. One such contextual factor may include incentives to authenticate medicines. The use of reimbursement codes within the 2D data matrix codes is hypothesized to help the authentication rate of medicines, a practice seen in the Belgian community pharmacy setting. There will be a legal mandate to authenticate medicines, however incentives such as reimbursement upon authentication may prove to augment not only authentication and detection rates but also legal compliance.

The results from this study should inform key opinion leaders, policy makers and technology manufacturers as to the limitations of medicines authentication technology and potential authentication technology improvements. Considering the limited evidence to support medicines authentication the outcomes of this study should also service decision makers in their discussions surrounding the selection of medicines authentication technology providers.

For peer review only - http://bmjopen.bmj.com/site/about/guidelines.xhtml

CONTRIBUTION STATEMENT

BN and DB were responsible for study conception, BN, DB, LR, SD and SC were responsible for planning, BN was responsible for data collection and scripting, BN and LR were responsible for data analysis. BN, DB, LR, SD and SC were responsible for the reviewing of this manuscript.

COMPETING INTERISTS

The content outlined herein represents the individual opinions of the authors and may not necessarily represent the viewpoints of their employers. D.A.B is an employee and/or stockholders in Aegate Ltd (Melbourn, UK) that is a provider of medicines authentication services. D.A.B. is also a stockholder in Translation Ventures Ltd. (Charlbury, Oxfordshire, UK) and IP asset ventures. D.A.B. is subject to the CFA Institute's Codes, Standards, and Guidelines, and as such, this author must stress that this piece is provided for academic interest only and must not be construed in any way as an investment recommendation. B.N is currently not, but has previously been a consultant of Aegate limited. Additionally, at time of publication, D.A.B. and the organizations with which he is affiliated may or may not have agreed and/or pending funding commitments from the organizations named herein.

FUNDING

This study was funded by Keele University, Oxford University and Aegate limited via an unrestricted educational grant.

DATA SHARING STATEMENT

Raw research data is available via Dryad

For peer review only - http://bmjopen.bmj.com/site/about/guidelines.xhtml

REFERENCES

1. Falsified medicines - European Commission [Internet]. [cited 2016 Oct 18]. Available from: http://ec.europa.eu/health/human-use/falsified_medicines/index_en.htm

2. Research C for DE and. Counterfeit Medicine [Internet]. [cited 2016 Sep 20]. Available from: http://www.fda.gov/Drugs/ResourcesForYou/Consumers/BuyingUsingMedicineSafely/CounterfeitMedicine/

3. Attaran A, Barry D, Basheer S, Bate R, Benton D, Chauvin J, et al. How to achieve international action on falsified and substandard medicines. The BMJ. 2012 Nov 13;345:e7381.

4. WHO | Substandard, spurious, falsely labelled, falsified and counterfeit (SSFFC) medical products [Internet]. [cited 2016 Sep 27]. Available from: http://www.who.int/mediacentre/factsheets/fs275/en/

5. Mackey TK, Liang BA. Improving global health governance to combat counterfeit medicines: a proposal for a UNODC-WHO-Interpol trilateral mechanism. BMC Med. 2013;11(1):233.

6. PSI-Inc.org [Internet]. [cited 2015 Aug 11]. Available from: http://www.psi-inc.org/incidenttrends.cfm

7. Hajjou M, Qin Y, Bradby S, Bempong D, Lukulay P. Assessment of the performance of a handheld Raman device for potential use as a screening tool in evaluating medicines quality. J Pharm Biomed Anal. 2013 Feb 23;74:47–55.

8. Mbinze JK, Sacré P-Y, Yemoa A, Mavar Tayey Mbay J, Habyalimana V, Kalenda N, et al. Development, validation and comparison of NIR and Raman methods for the identification and assay of poor-quality oral quinine drops. J Pharm Biomed Anal. 2015;111:21–7.

9. Sacré P-Y, Deconinck E, Saerens L, De Beer T, Courselle P, Vancauwenberghe R, et al. Detection of counterfeit Viagra® by Raman microspectroscopy imaging and multivariate analysis. J Pharm Biomed Anal. 2011 Sep 10;56(2):454–61.

10. Chen C, Zhang F, Barras J, Althoefer K, Bhunia S, Mandal S. Authentication of medicines using nuclear quadrupole resonance spectroscopy. IEEE/ACM Trans Comput Biol Bioinform. 2015;1–1.

11. Yeebo Y. The African Startup Using Phones to Spot Counterfeit Drugs [Internet]. Bloomberg.com. [cited 2016 Feb 17]. Available from: http://www.bloomberg.com/news/features/2015-07-31/the-african-startup-using-phones-to-spot-counterfeit-drugs

12. Book Excerpt: Fundamentals of EU Regulatory Affairs, Seventh Edition, Chapter 8: European Union Falsified Medicines Directive: Requirements and Implications for Multi-Stakeholder Healthcare Delivery | RAPS [Internet]. [cited 2016 Jan 25]. Available from: http://www.raps.org/Regulatory-Focus/RAPS-Latest/2015/12/18/23808/Book-Excerpt-Fundamentals-of-EU-Regulatory-Affairs-Seventh-Edition-Chapter-8-European-Union-Falsified-Medicines-Directive-Requirements-and-Implications-for-Multi-Stakeholder-Healthcare-Delivery/

For peer review only - http://bmjopen.bmj.com/site/about/guidelines.xhtml

13. COMMISSION DELEGATED REGULATION (EU) 2016/ 161 - of 2 October 2015 - supplementing Directive 2001/ 83/ EC of the European Parliament and of the Council by laying down detailed rules for the safety features appearing on the packaging of medicinal products for human use - http://eur-lex.europa.eu/legal-content/EN/TXT/PDF/?uri=OJ:JOL_2016_032_R_0001&from=EN [Internet]. [cited 2016 May 3]. Available from: http://eur-lex.europa.eu/legal-content/EN/TXT/PDF/?uri=OJ:JOL_2016_032_R_0001&from=EN

14. Directive 2011/62/EU of the European Parliament and of the Council of 8 June 2011 amending Directive 2001/83/EC on the Community code relating to medicinal products for human use, as regards the prevention of the entry into the legal supply chain of falsified medicinal productsText with EEA relevance - dir_2011_62_en.pdf [Internet]. [cited 2015 Jul 9]. Available from: http://ec.europa.eu/health/files/eudralex/vol-1/dir_2011_62/dir_2011_62_en.pdf

15. Upton F. H.R.3204 - 113th Congress (2013-2014): Drug Quality and Security Act [Internet]. 2013 [cited 2016 Feb 8]. Available from: https://www.congress.gov/bill/113th-congress/house-bill/3204

16. EFPIA - The European Medicines Verification Organisation (EMVO) signs framework agreements with service providers to establish blueprint systems [Internet]. [cited 2016 Oct 18]. Available from: http://www.efpia.eu/mediaroom/273/43/The-European-Medicines-Verification-Organisation-EMVO-signs-framework-agreements-with-service-providers-to-establish-blueprint-systems

17. Naughton, BD, Roberts, L., Dopson, S., Chapman, SR., Brindley DA.. Medicine Authentication Technology as a Counterfeit Medicine Detection Tool : A Delphi Method Study to Establish Expert Opinion on Manual Medicine Authentication Technology in Secondary Care. BMJ Open. (Pending editor decision)

18. Rowe G, Wright G. Expert Opinions in Forecasting: The Role of the Delphi Technique. In: Armstrong JS, editor. Principles of Forecasting [Internet]. Springer US; 2001 [cited 2015 Nov 9]. p. 125-44. (International Series in Operations Research & Management Science). Available from: http://link.springer.com/chapter/10.1007/978-0-306-47630-3_7

19. The Delphi Technique: Making Sense Of Consensus - v12n10.pdf [Internet]. [cited 2015 Nov 9]. Available from: http://pareonline.net/pdf/v12n10.pdf

20. Planning and Conducting Needs Assessments | SAGE Publications Ltd [Internet]. [cited 2016 May 25]. Available from: https://uk.sagepub.com/en-gb/eur/planning-and-conducting-needs-assessments/book5003

21. Adler M, Ziglio E. Gazing Into the Oracle: The Delphi Method and Its Application to Social Policy and Public Health. Jessica Kingsley Publishers; 1996. 270 p.

22. Predicting the Future: Have you considered using the Delphi Methodology? [Internet]. [cited 2015 Nov 9]. Available from: http://www.joe.org/joe/1997october/tt2.php/index.php

For peer review only - http://bmjopen.bmj.com/site/about/guidelines.xhtml

23. Group techniques for program planning : a guide to nominal group and delphi processes | Clc [Internet]. [cited 2015 Nov 9]. Available from: http://library.wur.nl/WebQuery/clc/347478

24. Ulschak FL. Human resource development: the theory and practice of need assessment. Reston Pub. Co.; 1983. 328 p.

25. Green P. The Content of a College-Level Outdoor Leadership Course. 1982 Mar [cited 2015 Nov 9]; Available from: http://eric.ed.gov/?id=ED276546

26. Shojania KG, Jennings A, Mayhew A, Ramsay C, Eccles M, Grimshaw J. Effect of point-of-care computer reminders on physician behaviour: a systematic review. CMAJ Can Med Assoc J. 2010 Mar 23;182(5):E216–25.

27. Dopson S, Fitzgerald L, Ferlie E. Understanding Change and Innovation in Healthcare Settings: Reconceptualizing the Active Role of Context. J Change Manag. 2008 Dec 1;8(3–4):213–31.

For peer review only
